# Fusion transcripts in normal human cortex increase with age and show distinct genomic features for single cells and tissues

**DOI:** 10.1038/s41598-020-58165-6

**Published:** 2020-01-28

**Authors:** Bharati Mehani, Kiran Narta, Deepanjan Paul, Anurag Raj, Deepak Kumar, Anchal Sharma, Lalit Kaurani, Subhashree Nayak, Debasis Dash, Ashish Suri, Chitra Sarkar, Arijit Mukhopadhyay

**Affiliations:** 1 Genomics and Molecular Medicine Unit, CSIR-Institute of Genomics and Integrative Biology, Mathura Road, New Delhi, 110020 India; 2 Academy of Scientific and Innovative Research (AcSIR), Delhi, India; 3 G.N. Ramachandran Knowledge Center for Genome Informatics, CSIR-Institute of Genomics and Integrative Biology, Mathura Road, New Delhi, 110020 India; 4 Department of Neurology, Neuroscience Centre, All India Institute of Medical Sciences, 110029 New Delhi, India; 5 Department of Pathology, All India Institute of Medical Sciences, New Delhi, 110029 India; 6 Department of Neurosurgery, All India Institute of Medical Sciences, New Delhi, 110029 India; 7 Translational Medicine Laboratory, Biomedical Research Centre, University of Salford, M5 4WT, Salford, United Kingdom

**Keywords:** Transcriptomics, Transcriptomics

## Abstract

Fusion transcripts can contribute to diversity of molecular networks in the human cortex. In this study, we explored the occurrence of fusion transcripts in normal human cortex along with single neurons and astrocytes. We identified 1305 non-redundant fusion events from 388 transcriptomes representing 59 human cortices and 329 single cells. Our results indicate while the majority of fusion transcripts in human cortex are intra-chromosomal (85%), events found in single neurons and astrocytes were primarily inter-chromosomal (80%). The number of fusions in single neurons was significantly higher than that in single astrocytes (p < 0.05), indicating fusion as a possible contributor towards transcriptome diversity in neuronal cells. The identified fusions were largely private and 4 specific recurring events were found both in cortex and in single neurons but not in astrocytes. We found a significant increase in the number of fusion transcripts in human brain with increasing age both in single cells and whole cortex (p < 0.0005 and < 0.005, respectively). This is likely one of the many possible contributors for the inherent plasticity of the adult brain. The fusion transcripts in fetal brain were enriched for genes for long-term depression; while those in adult brain involved genes enriched for long-term potentiation pathways. Our findings demonstrate fusion transcripts are naturally occurring phenomenon spanning across the health-disease continuum, and likely contribute to the diverse molecular network of human brain.

## Introduction

Biological systems utilize their built-in flexibility to respond to unknown situations that challenge their ‘fitness’ to adapt and respond. This flexibility usually increases with increased complexity and diversity of more recently evolved species. Human biology, in particular of the human brain, is one of the most diverse natural systems. At a molecular level, this flexibility is created and maintained by contributions from all layers of information - namely, DNA, RNA and Proteins, usually following an increasing order of diversity. We have earlier reported the extent of DNA level diversity and its possible role due to somatic single nucleotide variations in normal human brain^1^. Earlier studies have reported wide variety of DNA level diversity in neuron rich regions of normal human brain - ranging from whole chromosomes^2,3^, large-scale retro transpositions^4,5^, and copy number variations at the single neuron level^6^. We have also shown the diversity in the non-coding RNA of human brain attributed through the RNA editing mechanisms in miRNAs and their possible role in biological outcome^7^. In this study, we embarked on investigation of fusion transcripts in human brain - another possible mechanism by which the transcriptome can contribute to the diversity of complex systems.

Fusion transcripts have sequences from two or more genes, unlike conventionally spliced mRNA isoforms^8,9^. These fusions may arise at the genomic level by various structural rearrangements like deletion, duplication, inversion or translocation^10^ as well as at transcriptomic level by RNA-polymerase read-through^11,12^, cis- or trans-splicing^13^. Thus, investigation of transcriptomic analysis using massively parallel sequencing strategies provides an opportunity to capture all fusion transcripts – irrespective of their genomic or transcriptomic origins^14,15^.

Fusion transcripts and their encoded products were earlier perceived to be an aberration with negative outcome – owing to their abundance in cancers^16–18^. A few isolated studies have reported the existence of fusion RNAs within the normal human genome^19,20^.

In this study, we elucidated the landscape of fusion transcripts in the frontal cortex of human brain. For a comprehensive understanding, we have explored the transcriptome at the tissue level (cortex) as well as at the level of single neurons and astrocytes. Further, we analysed age related changes in fusion transcript of human brain. To our knowledge this is the most comprehensive study of fusion transcripts in normal human brain.

## Results

### Fusion transcripts in frontal cortex show a bias for intra-chromosomal events

We analyzed 59 whole-transcriptome datasets comprising of 49 prefrontal cortices (public domain) and 10 frontal cortices (sequenced in-house). By analyzing ~4.2 billion sequencing reads we identified 88 fusion transcripts representing 38 non-redundant events (Supplementary Table S1).

**Figure 1:**
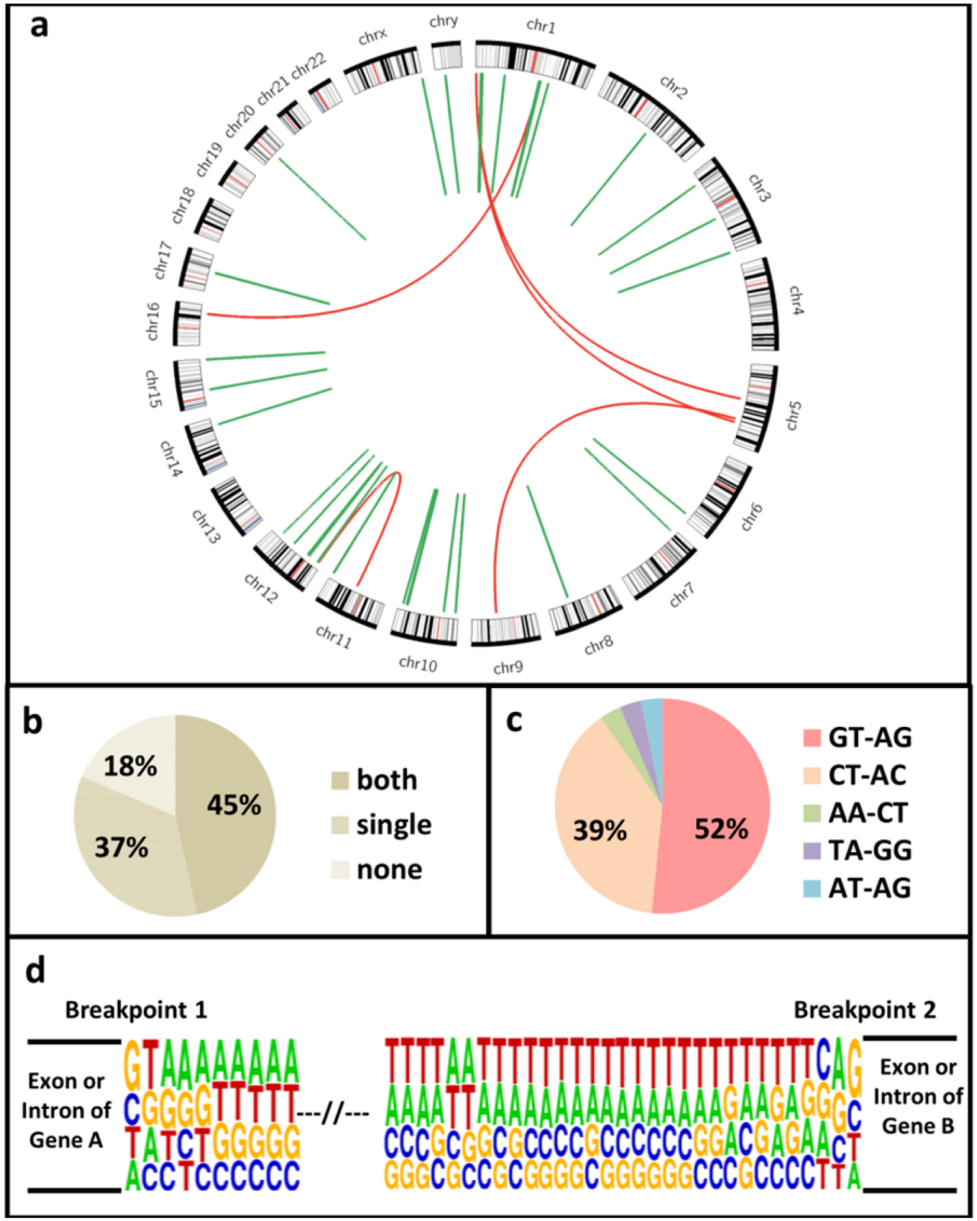
Fusion transcripts identified in human cortex. (**a**) Circos plot representing 38 unique fusions, where periphery of the plot represents different chromosomes and links denote fusion events. Red links are inter-chromosomal event involving genes from two different chromosomes and links that are in green are intra-chromosomal fusion between genes from same chromosome. (**b**) Percentage of fusion breakpoints containing exon boundaries from one, both or neither of their parent genes. (**c**) Percentages of fusion transcripts that use canonical splice site signature GT-AG, CT-AC, or another donor-acceptor sequential preference in brain tissues. (**d**) Preferred sequences around fusion breakpoint matching with the signature for canonical splicing.

We observed an enrichment of intra-chromosomal fusions amongst these events (87%, 33/38; Fig. 1a). Almost half of the events (47%, 18/38) were found at least twice, and 5 of them were in minimum 4 samples (Table 1). Analysis of the sequence context around the identified break-points revealed, 82% (31/38) of the events were fused at the exon boundaries from either one or both parental genes (Fig. 1b), and 91% (28/31) of them retained the canonical GT-AG splice sites (Fig. 1c). This indicated that a majority of the fusion transcripts, identified through our analysis, was formed by the classical splicing machinery – albeit joining fragments from 2 different mRNAs.

**Table 1:** Fusion transcripts identified in 59 brain samples. The asterisk (*) represents events retaining exon boundaries from both of its partner genes.

Gene left	Gene right	Chr left	Cord left	Chr right	Cord right	Recurrence	Dinucleotide preference	Left exon intact	Right exon intact
KANSL1*	ARL17A*	chr17	44171925	chr17	44430295	10	GT-AG	yes	yes
KANSL1*	ARL17A*	chr17	44171925	chr17	44648234	9	GT-AG	yes	yes
ENSG00000212127*	PRH1-PRR4*	chr12	11126253	chr12	11001005	5	GT-AG	yes	yes
ENSG00000227733*	HYDIN*	chr1	146126403	chr16	71196632	4	GT-AG	yes	yes
FAM78B	ENSG00000229588	chr1	166135290	chr1	166304564	4	GT-AG	no	yes
FLJ39739	NBPF9	chr1	147917467	chr1	144676872	3	CC-TA	no	no
CCDC7*	C10orf68*	chr10	32854485	chr10	32873231	3	CT-AC	yes	yes
ABLIM1	ENSG00000228484	chr10	116361719	chr10	116527819	3	CT-AC	yes	no
LOC100129961*	ENSG00000224043*	chr2	135635089	chr2	135493150	3	GT-AG	yes	yes
ENSG00000225065*	NCOA6*	chr20	33302393	chr20	33303168	3	GT-AG	yes	yes
ENSG00000243795	C3orf17	chr3	112862700	chr3	112738552	3	CT-AC	no	yes
ENSG00000236537*	TULP4*	chr6	158733082	chr6	158735299	3	CT-AC	yes	yes
LOC729852	ENSG00000233108	chr7	7841373	chr7	8007329	3	GT-AG	yes	no
ENSG00000218328	KAZN	chr1	14507086	chr1	14925478	2	GT-AG	yes	no
PDE4DIP	FLJ39739	chr1	145013720	chr1	147931723	2	CT-AC	no	yes
CCDC7*	C10orf68*	chr10	32832227	chr10	32873231	2	CT-AC	yes	yes
HSPA12A	ENO4	chr10	118466795	chr10	118609076	2	CT-AC	yes	no
ENSG00000231121*	NAV3*	chr12	77966045	chr12	78334098	2	GT-AG	yes	yes
ENSG00000230021	PCBD2	chr1	564469	chr5	134261453	1	AC-TG	no	no
ENSG00000198744	DHFR	chr1	570067	chr5	79946838	1	GC-CA	no	no
MTOR	UBIAD1	chr1	11316632	chr1	11334116	1	CT-AC	no	yes
ENSG00000231485*	JAK1*	chr1	65532310	chr1	65352023	1	GT-AG	yes	yes
PFKFB3*	LOC399715*	chr10	6268327	chr10	6368508	1	GT-AG	yes	yes
ENSG00000249456	ZRANB1	chr10	126628942	chr10	126631875	1	CT-AC	no	yes
ALG1L9P	FAM66C	chr11	71518527	chr12	8346798	1	GT-AG	no	yes
OPCML*	NTM*	chr11	132812820	chr11	132081915	1	GT-AG	yes	yes
ENSG00000245482	ALG10B	chr12	34185072	chr12	38720271	1	CT-AA	no	no
ACAD10	MAPKAPK5	chr12	112182444	chr12	112308981	1	AA-CT	yes	yes
PSMC1	ENSG00000205533	chr14	90738533	chr14	90739625	1	AG-CC	no	no
ENSG00000186031*	FMN1*	chr15	33445248	chr15	33300275	1	GT-AG	yes	yes
LRRK1	CHSY1	chr15	101590983	chr15	101775286	1	CT-AC	no	yes
THRB	THRB-AS1	chr3	24378861	chr3	24536623	1	CT-AC	yes	no
ENSG00000249598	SDHAP1	chr3	195685882	chr3	195686956	1	GT-AG	no	yes
ENSG00000250859	HNRNPK	chr5	126847434	chr9	86585718	1	AT-AG	no	yes
ENSG00000236537*	TULP4*	chr6	158703294	chr6	158735299	1	CT-AC	yes	yes
CPQ	TSPYL5	chr8	97919555	chr8	98287867	1	CT-AC	no	no
CLIC2	ENSG00000224216	chrX	154563678	chrX	154564556	1	TA-GG	yes	yes
TTTY14	NCRNA00185	chrY	21239153	chrY	21039090	1	GT-AG	no	no

**Figure 2:**
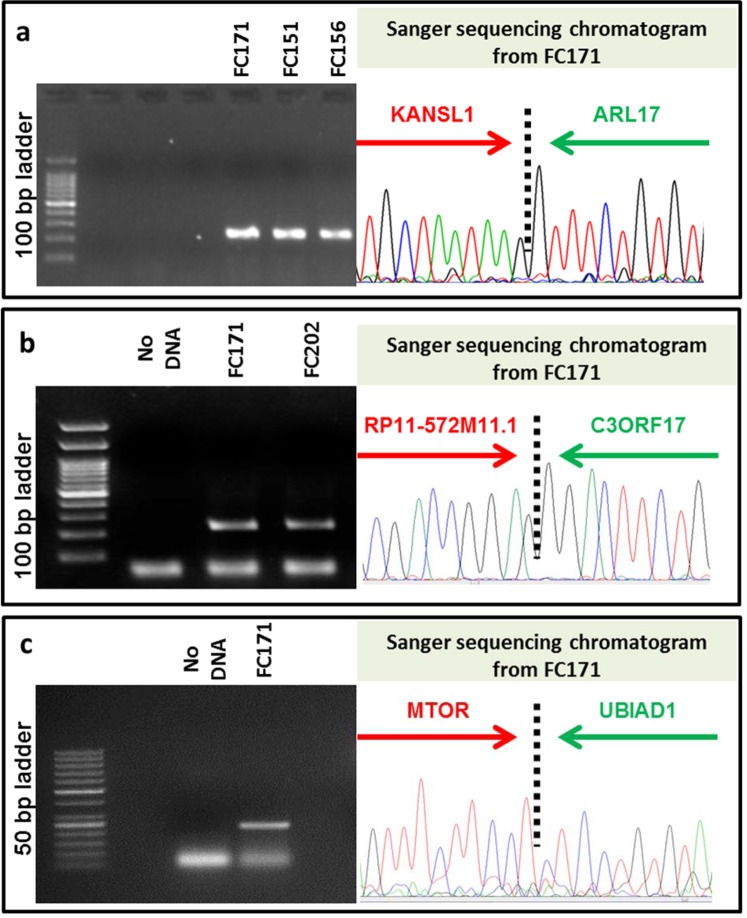
Fusion specific PCR product was run on 2% agarose gel and further confirmed by using Sanger sequencing. Validation of (**a**) *KANSL1-ARL17* fusion was validated in FC171, FC151 & FC156 with a product size of 239 bp; (**b**) *C3ORF17-RP11-572M11.3* fusion in FC171 & FC202 with a product size of 227 bp and (**c**) *mTOR-UBIAD1* fusion in FC171 with a product size of 236 bp. Strategy used to design the fusion specific primer is depicted in the Supplementary Fig. S7.

We used the available frontal cortex (FC) samples for experimental validation of the identified fusions. Based on the recurrence and supporting reads, 3 selected fusion events were validated using fusion specific PCR followed by Sanger sequencing. They include, (i) two recurring events, namely, KANSL1-ARL17A (Fig. 2a) (also see later) and RP11-572M11.1-C3ORF17 (Fig. 2b) along with (ii) a private event: MTOR-UBIAD1 (Fig. 2c). MTOR-UBIAD1 was further quantified by qPCR and was found to have lower expression (~0.3 fold) with respect to MTOR – one of the parent genes (Supplementary Fig. S1).

### Fusion transcripts in single cells are rich in inter-chromosomal events

We used single cell transcriptome data from human brain to resolve the tissue level data with increased resolution. For this we considered publically available transcriptomic libraries from 131 neurons and 62 astrocytes collected from 7 different healthy adult brains. Upon analyzing 500 million reads (minimum 1 million reads per cell, Supplementary Fig. S2), a total of 912 non-redundant fusion events were identified (Supplementary Tables S2 and S3).

**Figure 3:**
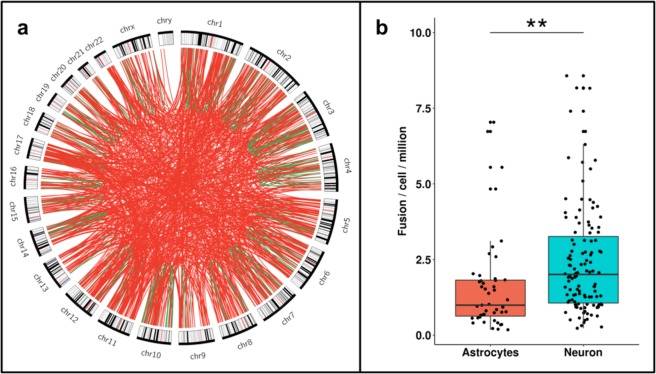
Fusion transcripts identified in single cells from adult human brain. (**a**) Circos plot represents 911 unique fusions and signifies 82% (749/911) of the identified events are Inter-chromosomal. Here, periphery of the plot represents different chromosomes and links denote fusion events. Red links are inter-chromosomal event involving genes from two different chromosomes and links that are in green are intra-chromosomal fusion between genes from same chromosome. (**b**) Neurons (blue) harbor significantly higher number of fusions compared to astrocytes (red) (one tailed Wilcoxon p value < 10^−3^ marked by **), where boxplot represents number of fusion transcripts identified in each cell. Horizontal axis denotes brain cells while vertical axis represents number of fusion transcripts normalized with total data generated in that cell.

Interestingly, fusion transcripts identified in single cells demonstrated a clear bias for inter-chromosomal events with 82% (749/912) fusions involving genes from two different chromosomes (Fig. 3a) – which was in contrast to our findings for the cortex samples. Fusion transcripts from single cells revealed a smaller fraction that harbor exonic boundaries from either of the two partner genes (28%, 251/911) and only ~33% (82/251) of them harbor the canonical GT-AG site (Supplementary Fig. S3).

Three recurring fusions were identified in single neurons which were also found to recur in cortex samples (Supplementary Fig. S4). In contrast, single astrocytes had all private events and none of them were common with the events found in the cortex samples.

Surprisingly, we found significantly higher (one tailed Wilcoxon p value < 0.005) number of fusions in neurons when compared to the astrocytes (Fig. 3b). Simultaneously, expression analysis revealed that 87% of the genes harboring fusion in neurons, have a considerable expression (FPKM > 1) in astrocytes (Supplementary Fig. S5A) and only 27% (462/1726) of them were differentially expressed. The expression analysis indicates the difference in fusion load is not due to absence of the expression of genes that are involved in fusion.

### Adult brain harbor more fusion transcripts than fetal brain

To ascertain any association between the fusion burden and aging brain we considered another data set encompassing brain transcriptomes from two distinct age groups. Towards this we analyzed a total of ~2 billion RNA sequencing reads from Dorso Lateral Frontal Cortices (DLFC) derived from 16 adult and 8 newborns together with 131 and 110 single neurons derived from 7 adult and 5 fetal brains respectively. We identified 63 unique fusions in 16 adult brains while 10 fusions in fetal brain. Similarly, 724 fusion events were observed in adult neurons and 302 in fetal neurons (Supplementary Tables S3–S6).

**Figure 4:**
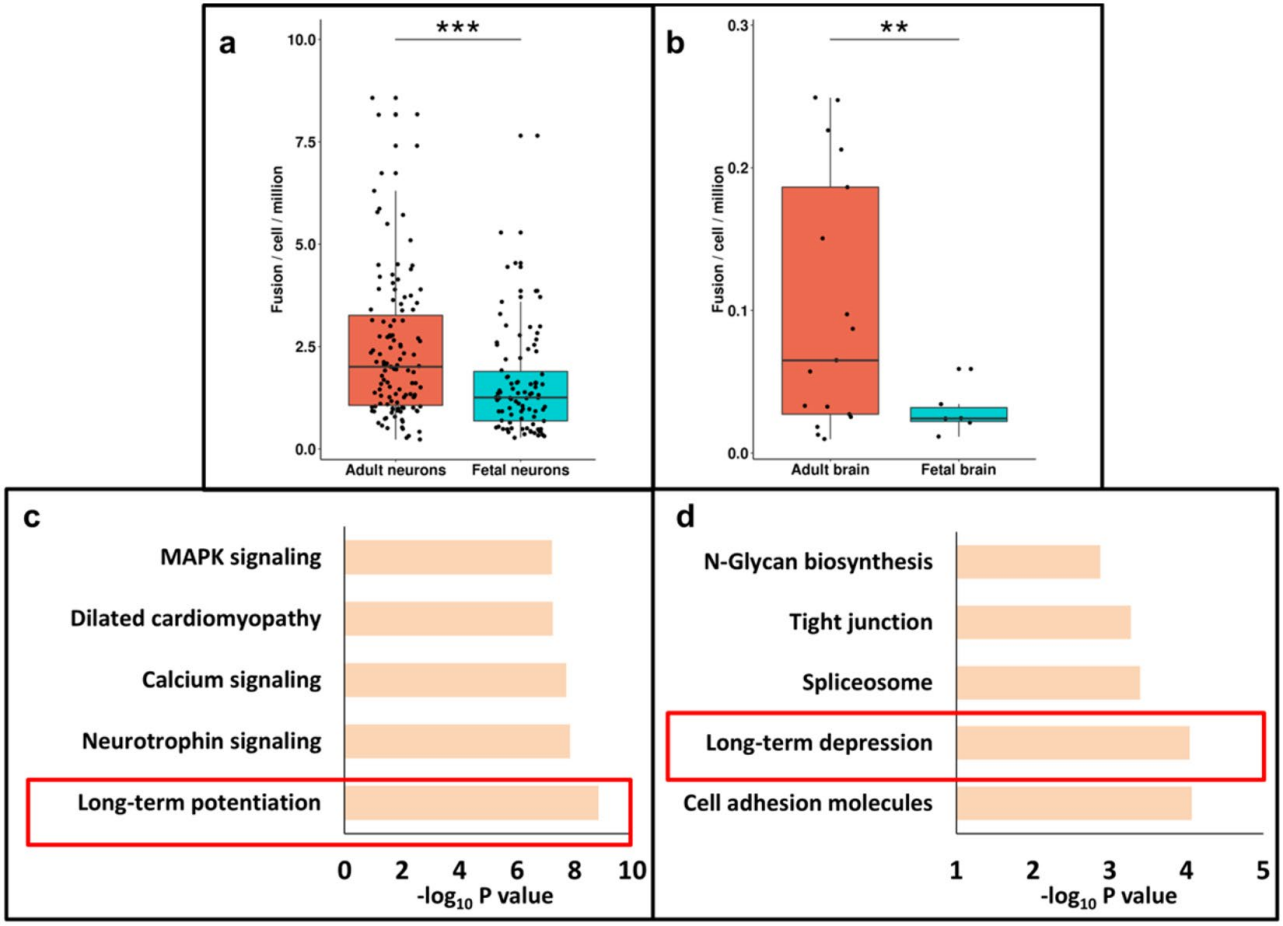
Boxplots represent fusion transcripts are enriched in adult brain compared to the fetal brain. (**a**) In case of single cells, adult neurons (red) harbor significantly higher number of fusions with respect to fetal neurons (blue) (one tailed Wilcoxon p value < 10^−4^ is marked by ***). (**b**) Similarly, bulk tissue from adult brain (red) also demonstrated higher number of fusions compared to fetal (blue) (one tailed Wilcoxon p value < 10^−3^ is marked by **) where horizontal axis denotes brain samples while vertical axis represents number of fusion transcripts normalized with total data generated in a samples. Pathway enrichment analysis of genes harboring fusions has revealed enrichment for genes implicated in processes involved in synaptic plasticity. In the figure the horizontal axis shows the negative logarithm of FDR corrected p-value while vertical axis has different biological processes. Top biological processes that were significantly enriched in adult brain (**c**) and fetal brain (**d**) (both from tissue and single cells).

Strikingly, fusion transcripts were found to be higher (~2 times) in adult brain compared to the fetal brain, be it a cell or a tissue (one tailed Wilcoxon p value < 0.05) (Fig. 4a,b). Concurrently, expression analysis demonstrated that 88% of the genes harboring fusion in adults also have a considerable expression (FPKM > 1) in fetal brain (Supplementary Fig. S5B,C), with only 17% (287/1726) of them were differentially expressed between them.

To know the functional significance of these genes that undergo fusion, we also performed their enrichment analysis using GSEA. Genes harboring fusion demonstrated a significant enrichment for the long-term potentiation pathway in adults while in fetal there was a significant representation for long-term depression pathway (both with p value < 0.00001) (Fig. 4c,d).

### Translational ability of fusion transcripts

To test the translational ability of these fusion events we performed in-silico translation around the junction by translating them into six reading frames. These putative fusion peptides were first subjected to in-silico digestion with trypsin followed by MS identification. We used publically available MS/MS data from human cortex to map our digested peptides and identified 214 putative translated junctions. Among them 23% (49/214) were found in multiple proteomes (Supplementary Table S7) and 15% (31/214) retained the reading frame from either of their parent gene (Table 2). One of the best hits was the *GDI2-FAM208B* junction peptide which retained the reading frame from both of its parent genes.

**Table 2:** Table represents putative translated junction for identified fusions a reading frame of their parent genes. Highlighted events are identified in multiple proteomics samples.

GeneA	GeneB	Putative translated fusion peptide	Junction peptide from MS data	GeneA and its reading frame	GeneB and its reading frame
GDI2	FAM208B	IIVQNGKVIGVKSEGENLLRKGGHTEIEPQHF	VIGVKSEGENLLR	intact	intact
ENSG00000225302	HSPA12A	AKNKMKCDSRWEIAASETAPTSAYSSPARSLGD	WEIAASETAPTSAYSSPAR	intact	intact
ENSG00000212127	PRH1-PRR4	LPAGTCCIYSRVEVFTDVNYEDFTFTIPGKSQ*	LPAGTCCIYSRVEVFTDVNYEDFTFTIPGK	intact	intact
MAP1B	DDX3×	EKIERTTKSPSDSGYSYRPCVVYGGADIGQQIR	SPSDSGYSYRPCVVYGGADIGQQIR	intact	intact
YLPM1	ZNF207	MVPPYQGGPPRPPMGMRPPPPLPPPPPVIKPQT	PPYQGGPPRPPMGMRPPPPLPPPPPVIKPQT	intact	intact
KLHL8	SMARCAD1	LLRFYENGELCDVTLKLIESTSTMDGAIAAAL	FYENGELCDVTLKLIESTSTMDGAIAAAL	intact	intact
SLC7A5P2	PTPRD	CPVPEEAAKLVACHSVPPPRFTRTPVDQTGVSG	LVACHSVPPPRFTR	intact	intact
CSAD	ARHGEF12	CA*WKEMSIPLKSSFLLWQDLICRMAASVKEQS	SSFLLWQDLICR	intact	no
GABRB2	ZNF451	MNIDIASIDMVSEVNMNVTVMITWVPKILQ*E	NIDIASIDMVSEVNMNVTVMITWVPK	intact	no
PPP2R4	NPAP1	IDTSDMNTTPPSKTVILQSGQGLRLALV*ESCF	IDTSDMNTTPPSKTVILQSGQGLR	intact	no
ACAD10	MAPKAPK5	QGDLMTPQFTPYYVAPQGKQAPHMRNKLES*PN	QGDLMTPQFTPYYVAPQGKQAPHMR	intact	no
EIF4G3	FRMPD2	RSILNKLTPQMFNQLMKHLPGARHYSRPPSMLR	LTPQMFNQLMKHLPGAR	intact	no
OPCML	NTM	KAMDNVTVRQGESATLSISQNCRDFFRYLH**R	QGESATLSISQNCRDFFR	intact	no
MALAT1	MLL2	RCEPPRLAGSPFFLTPTNLPSTISPGLGSLPSK	CEPPRLAGSPFFLTPTNLPSTISPGLGSLPSK	intact	no
WDR6	CBFA2T3	SGPGGVVACLEISAAPSGICVCGVARGLASVRV	SGPGGVVACLEISAAPSGICVCGVARGLASVR	intact	no
WDR48	AATF	LFKDKGGPEFSSALKNMNLGGLLLQALLEYWPR	GGPEFSSALKNMNLGGLLLQALLEYWPR	intact	no
GRAMD2	HSP90AA1	PEGLKGEEIKKCGREGVNLWVVEQKLSYT*KKT	CGREGVNLWVVEQK	intact	no
SYNE1	SSBP1	PPKEPMDMEAQLMDCQASETWHINM*KRGKLKR	PPKEPMDMEAQLMDCQASETWHINM	intact	no
NUMB	ARHGAP24	YLPGLSKPLPYCEELFYILFSMQVKTHNIDFIN	YLPGLSKPLPYCEELFYILFSMQVKTHNIDFIN	intact	no
SH3PXD2A	BCLAF1	MGRASHLVHDMQRLPEDQEALDYFSDKESGKQK	ASHLVHDMQRLPEDQEALDYFSDK	no	intact
ZNF292	ARNT2	SQGLSIQSLRNTIGLLIHIFNKHNDKHKAHLIR	SQGLSIQSLRNTIGLLIHIFNK	no	intact
NCKAP1	KIAA1109	RSIVGMTMYNQATQEIALAADHHSKHEAQRHFL	SIVGMTMYNQATQEIALAADHHSK	no	intact
USP11	ATL2	ARVGENVHCGPAKAGENYEDDDLVNSDEVMKK	VGENVHCGPAKAGENYEDDDLVNSDEVMK	no	intact
DPP6	MLLT3	RRRTWTVSILAWLCTPEKPSKDSREHKSAFKE	TWTVSILAWLCTPEKPSKDSR	no	intact
USP34	DDHD1	MRKCVVQLCQGGWLIGQKMDQGRIIKNTAM*VL	CVVQLCQGGWLIGQKMDQGR	no	intact
FAM185A	FBXL13	HCQKNHTAKTSPNSWPTGNITLQSKMGNITVGM	TSPNSWPTGNITLQSKMGNITVGM	no	intact
NMD3	MKLN1	ADDYNCKQC*RLALGSEDGLDFYYSSKQHAQKM	LALGSEDGLDFYYSSK	no	intact
ENSG00000178440	TIMM23	TGEVLRRSYTKGSKVLDTDEFILPTGANKTRG	GSKVLDTDEFILPTGANK	no	intact
ENSG00000147421	PCDH9	QVMKWKASLTAVISSDTLISHPLPLVQPQDEF	ASLTAVISSDTLISHPLPLVQPQDEF	no	intact
GRIA4	ENSG00000234873	LDQ*QKQVTHQGTGQPWSWSWLVLLCPPPRGE	QVTHQGTGQPWSWSWLVLLCPPPRGE	no	intact
FAM226B	NAP1L2	SSLFSSPVASMSSSSSSSFTATERNWGGCHSLW	SSLFSSPVASMSSSSSSSFTATER	no	intact

### Topology of the chromosomes inside a cell and fusion transcripts

**Figure 5:**
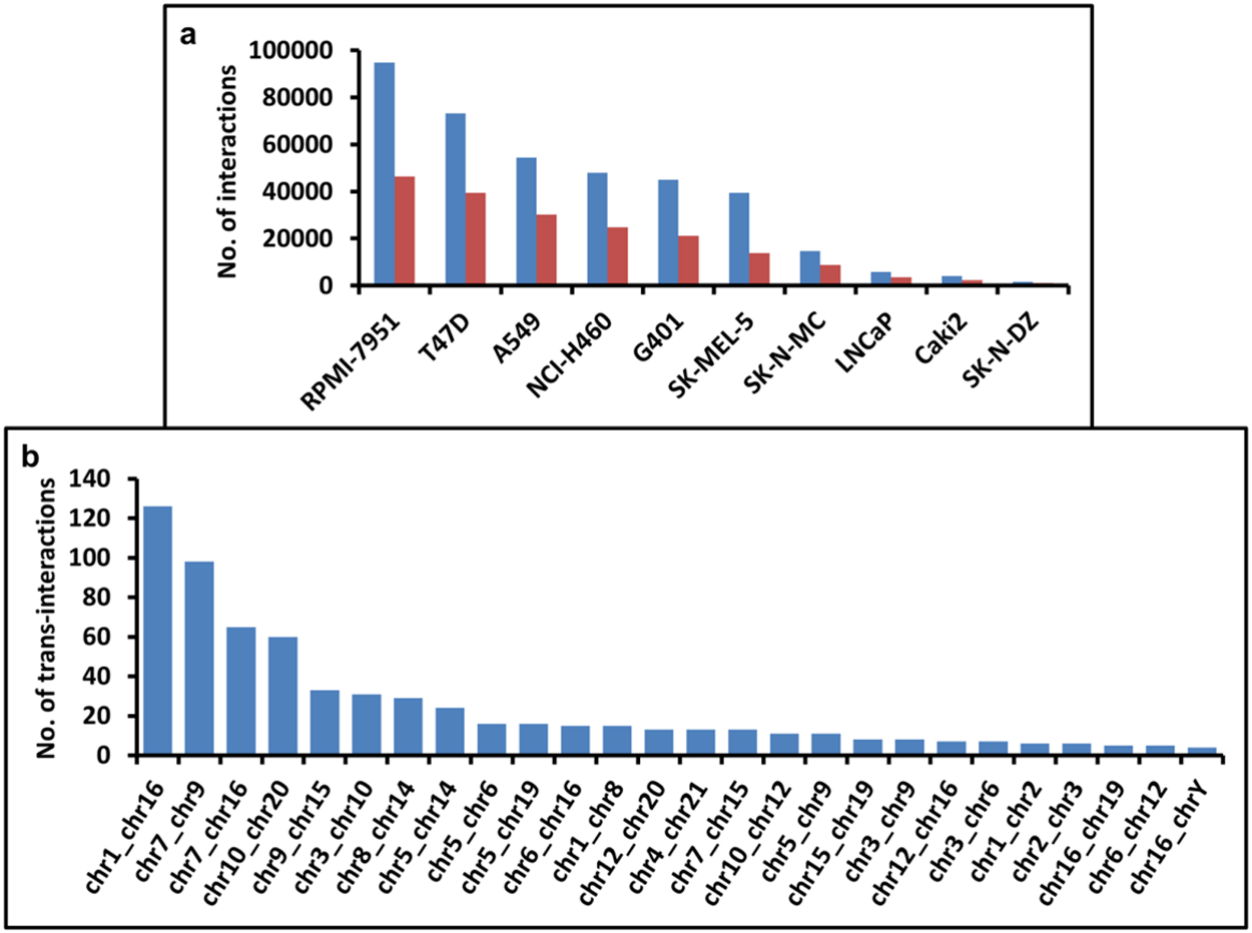
Spatial proximity might be a possible trigger for fusion transcripts. (**a**) Chromatin interaction map identified in 10 human cell lines. Horizontal axis in the plot represents different cell lines and vertical axis represents number of chromatin interaction identified in each cell line at 40 Kb resolutions. Blue bars represent raw numbers of interactions while that are in red are significant interaction identified with Z score > 1.96 in each cell line. (**b**) Chromosome pairs assisting chromatin interactions and their numbers. Horizontal axis represents different chromosome pairs and vertical axis represents number of trans-interactions identified.

To test if fusions can occur between spatially proximal regions of the genome, we also performed an unbiased chromatin interaction mapping. To test the same, we used ENCODE Hi-C data from 10 different human cell lines. This set of analysis led us to identify 32190 significant genome wide chromatin interactions (Z score > 1.96) reported in at least 2 cell lines at a 40 kb resolution (Fig. 5a and Supplementary Table S8). This set of analysis demonstrated frequent cis-chromatin interactions within chr 17 and maximum number of trans-interactions between chr 1 and chr 16 (Fig. 5b).

By comparing fusion breakpoints with the Hi-C interaction maps of three-dimensional chromosome conformation led us to detect nine fusion partners located in broad chromatin domains that are spatially proximal in normal cell nuclei (Table 3). These events involve an interesting inter-chromosomal ENSG00000227733-HYDIN fusion involving ENSG00000227733 and HYDIN genes from chromosome 1 and 16 respectively. ENSG00000227733-HYDIN fusion was one of the recurring events identified in both single neurons as well as in brain tissues indicating its neuronal preference. Co-occurrence of Hi-C interaction map around its breakpoint suggests spatial proximity could be an important trigger to facilitate this fusion between the two distantly located genomic loci.

**Table 3:** Fusion partners that are also in physical proximity.

Fusion_Pair	Fusion breakpoint1	Fusion breakpoint2	Chromatin Interacting locus1	Chromatin Interacting locus2	Chromatin Interaction identified in cell line
ENSG00000249456_ZRANB1	chr10:126631875	chr10:126628942	chr10:126640000-126680000	chr10:126600000-126640000	A549, NCI-H460, RPMI-7951, T47D
ZFYVE9_ENSG00000154222	chr1:52815403	chr1:52805352	chr1:52800000-52840000	chr1:52760000-52800000	A549, RPMI-7951, SK-MEL-5, T47D
ENO2_ATN1	chr12:7033739	chr12:7031013	chr12:7040000-7080000	chr12:7000000-7040000	A549, RPMI-7951, SK-MEL-5, G401
ENSG00000225065_NCOA6	chr20:33303168	chr20:33302393	chr20:33320000-33360000	chr20:33280000-33320000	NCI-H460, RPMI-7951, T47D, G401
FAM177A1_PPP2R3C	chr14:35557754	chr14:35548998	chr14:35560000-35600000	chr14:35520000-35560000	A549, NCI-H460, G401
ENSG00000227733_HYDIN	chr1: 146126403	chr16:71196632	chr1:146100000-146140000	chr16:71160000-71200000	NCI-H460, T47D, G401
GDI2_FAM208B	chr10:5798575	chr10:5815804	chr10:5800000-5840000	chr10:5760000-5800000	A549, Caki2
SLC7A6_SLC7A6OS	chr16:68338040	chr16:68333926	chr16:68320000-68360000	chr16:68280000-68320000	A549, RPMI-7951
RENBP_SSR4	chrX:153062912	chrX:153208306	chrX:153160000-153200000	chrX:153080000-153120000	A549, SK-N-MC

### KANSL1-ARL17A transcript and its status in GBM

KANSL1-ARL17 fusion was one of the most recurring events comprising of first three exons from KANSL1 and last three exons from ARL17. It was identified in ~20% (12/59) of normal brain tissues (Supplementary Table S1). CNV signature around its junction from our analysis signifies its genomic origin and it is also a well-documented DNA level event in multiple cancers (Table 4).

**Table 4:** Fusion transcripts having CNV signature around their breakpoints.

Sample	Gene A	Chr Gene A	Cord Gene A	Gene B	Chr Gene B	Cord Gene B	chr	CNV satrt	CNV end	CNV state	CNV data source
156 fc	*KANSL1*	chr17	44171925	*ARL17A*	chr17	44430295	chr17	44757175	44782177	state2,cn = 1	660quad microarray
156 fc	*KANSL1*	chr17	44171925	*ARL17A*	chr17	44648234	chr17	44757175	44782177	state2,cn = 1	660quad microarray
171 fc	*MTOR*	chr1	11316632	*UBIAD1*	chr1	11334116	chr1	12057354	12062160	dup	Exome Sequencing

**Figure 6:**
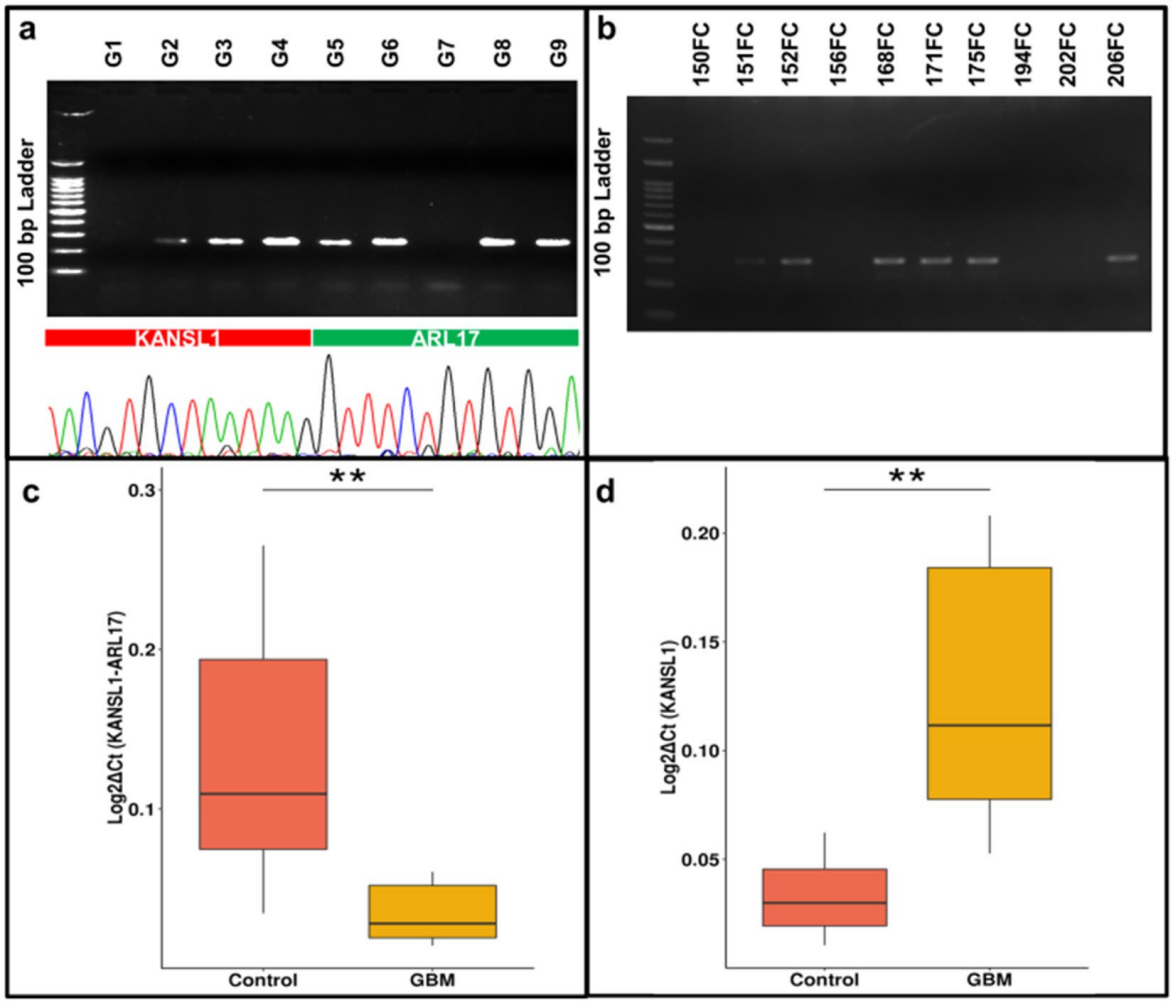
Validation of *KANSL1-ARL17* fusion in 7/9 GBM. Fusion specific PCR products (239 bp) were ran on 2% gel with 100 bp ladder and further confirmed by Sanger sequencing. Lower panel denotes a representative electropherogram generated by using cDNA from GBM_8163 which was ran on lane 4 of the agarose gel. Rest other electropherograms are shown in the supplementary file as Supplementary Fig. S6. (**b**) Confirmation of H1 β duplication haplotype in 6/10 in-house samples (FC). PCR products (307 bp) were run on 2% gel with 100 bp ladder. Relative expression of fusion transcript along with its parent genes was checked in GBM compared to controls. We performed real time PCR using (**c**) *KANSL1-ARL17* fusion, (**d**) *KANSL1* primers with cDNAs from 6 in-house normal brains along with 6 GBM samples. In both the boxplots, vertical axis represents Log_2_ΔCt calculated using the expression of B2M. We also performed the same set of experiment using fusion specific primers for ARL17 and depicted in the Supplementary Fig. S8. Strategy used to design the fusion specific primer is depicted in the Supplementary Fig. S7.

To test the status of this particular fusion in disease condition we used a most malignant brain tumor i.e. Glioblastoma multiforme (GBM). By performing junction specific PCR, we validated the same event in 7 out of 9 (78%) GBM samples and further confirmed it by Sanger sequencing (Fig. 6a and Supplementary Fig. S6). The large difference in frequency of this fusion event in GBM and normal brain led us to investigate the 17q21.31 locus, which harbours both the parent genes (*KANSL1* and *ARL17)*.

17q21.31 is a cryptic locus and known to have two different haplotypes along with their subtypes^21^. H1β duplication is the only subtype capable to produce *KANSL1-ARL17* fusion. We confirmed H1β duplication in our in-house normal brain samples which were observed to have an allele frequency of 70%, (7/10) as it was detected in the case of GBM samples (Fig. 6b). These results highlight the prominence of H1β duplication in south Asian population with an allele frequency close to 70%.

The expression of the KANSL1-ARL17 fusion transcript and its parent genes were also assessed with Real-Time PCR between GBM and our in-house normal brain tissues. Interestingly, KANSL1-ARL17 fusion transcript was found to be down regulated (5 ×) in GBM while it was up-regulated (5 ×) to the same extent in normal brain when compared to its parent gene (KANSL1) (Fig. 6c,d). These results indicate fusion product might have different function compared to its parent genes. Likewise, it’s not the presence rather the expression levels of the KANSL1-ARL17 fusion can be linked with cancer etiology.

## Discussion

The life span and ‘fitness’ of an organism is the sum of deleterious changes and counteracting repair and maintenance mechanisms that trigger in response to stimuli^22^. This is not only true for whole organisms but also true at the levels of tissues, organs and individual cells. Recent surge of next generation sequencing data has revealed that in the human brain this diversity is achieved and maintained not only by the inherited set of variations but also by the variability introduced *de novo* at the somatic level for both DNA and RNA^23–25^. Fusion transcripts are one of many possible ways to create and maintain the much needed diversity – especially in post-mitotic cells like human neurons. Fusion transcripts are now widely reported in the literature albeit with a bias towards their presence and function in diseases – especially for their oncogenic role in human cancers. However, these transcripts can have a big influence on the normal phenotypic outcome. It has been shown for fusion transcripts that the fusion event when created at the RNA level provide a growth advantage while the same event resulting from a DNA level translocation may lead to cancer^13^. The underlying explanation for this observation is likely due to the obligatory (and hence more) expression of the fusion RNA when it is formed at the DNA level. This suggests a functional continuum of the fusion transcripts, where the same events in moderation can provide an advantage while an excess is detrimental. The spectrum of fusion transcripts in normal human tissues and to what extent they are functionally relevant is an unanswered question. We embarked on this study in an attempt to address this issue focused to the normal human brain, particularly the cortex.

Our analysis showed the organization and distribution of the fusion transcripts are unique and distinct between the tissue and the single cells. At the tissue level we found more recurrence and majority of the events formed between two genes residing on the same chromosome. Notably, our analysis has identified KANSL1-ARL17 fusion as a recurring event in normal human cortex. Contrasting expression pattern between the KANSL1-ARL17 fusion and its parent gene, KANSL1 in GBM compared to the normal brain warrants further investigation, as it suggests their potential role in cancer.

On the other hand, the spectrum of fusion transcripts occurring from the single neurons and astrocytes show minimal or no recurrence and the vast majority formed by fusion of two transcripts coming from different chromosomes. Recurrence is an important factor to estimate their abundance. In our case, majority of our events were non-recurring between cells which might signify their low abundance or they may be false positive generated by template switching of reverse transcriptase during RT-PCR^26,27^ or possibly a mix of both. Further, a complete lack of redundancy in the single astrocytes was striking and cannot be explained. Further research on the same source of tissue and single cells might be able to shed light on these observations.

We found significantly higher number of fusions in neurons compared to the astrocytes. This observation, together with a considerably higher number of fusions in adult brain compared to the newborns – indicate a role of fusion transcripts in maintaining required diversity in neurons with restricted regenerative capabilities and age-dependent decline in turnover rate of the cells^28,29^. Genes harboring fusion demonstrated a significant enrichment for long-term potentiation (LTP) and long-term depression (LTD) pathways in adult and fetal brain respectively. Both of these pathways in conjunction provide neuronal synaptic plasticity that undergo age-related alterations^30–33^. Results from our analysis suggest aging leading to a substantial fusion load, which might affect the neuronal synaptic function. Evidence for their corresponding junction peptides further support their biological relevance. Later on we also identified the inter-chromosomal proximity between ENSG00000227733 and HYDIN genes from chromosome 1 and 16, respectively. Co-occurrences of chromatin interaction maps with these fusion breakpoints suggest spatial proximity can be one of the possible triggers.

In summary, our genome-wide analysis establish fusion transcripts are naturally occurring phenomenon that span the health-disease continuum of the human brain.

## Materials and Methods

### Sample collection

Frontal cortexes (Grey matter) were procured from post-mortem samples of road accident victims collected from NIMHANS Brain Bank, Bangalore, India. GBM samples were obtained from AIIMS, New Delhi, India. The samples were collected according to the Helsinki Declaration and the ethical review board of All India Institute of Medical Sciences, Delhi, India approved the project. Diagnosis and grading of tumor samples were done as per 2007 WHO classification. The details of the in-house samples used in the study are provided in Supplemental Table S9 and S10. Informed consent were obtained from the GBM patients or from their family members (next of kin). For the frontal cortex (post-mortem) samples obtained from the brain bank, these samples were already covered under the ethical approval obtained by the Brain bank.

### RNA isolation, library preparation and RNA sequencing

Total RNA was isolated by using miRvana miRNA isolation kit (Ambion, USA) as per the manufacturer’s instructions. RNA Libraries were prepared using Illumina’s TruSeq total RNA Sample preparation for 6 FCs and poly-A enriched protocol for rest of the 4 FCs following the manufacture’s protocol. Cluster generation and sequencing was done on Illumina HiSeq. 2000 using standard Illumina sequencing workflow. The in-house sequencing data is deposited at the sequence read archive (SRA ID: SRP045655).

### DNA isolation, library preparation and exome sequencing

DNA isolation was done by using Omniprep Genomic DNA isolation kit (G-Biosciences, USA) as per manufacturer’s protocol. Exome capture was done using Illumina TruSeq Exome capture kit. 100 base pair paired end sequencing was done using Illumina HiSeq. 2000 (Ilumina, USA). The exome sequence data is also deposited at the sequence read archive (SRA ID: SRP045655).

### Identification of candidate fusion transcripts

Raw data was checked for per base quality score and reads having 80% bases with phred quality score 30 and greater were only be considered for downstream analysis. Fusion transcripts were identified using the published pipeline^34^ with the default parameters. Briefly, the quality filtered reads were aligned using Tophat (version 2.0.5) against transcriptome (UCSC hg19 annotations) and genome (hg19) both with 2 mismatches. Discordant reads were used to identify potential fusion candidates using its fusion-search module. Tophat-fusion-post was further used to filter out events supported by minimum 5 supporting reads. The software and detailed description is available at < https://ccb.jhu.edu/software/tophat/fusion_tutorial.shtml >. The same pipeline was used for both in-house and publicly available dataset for calling fusion transcripts.

### Fusion transcripts associated with CNVs

To determine if the origin of the detected fusions were genomic, we explored signatures for copy number variation around fusion breakpoint Genome-wide CNV calling was performed using two different platforms: exome sequencing and illumina’s 660 quad microarray for 4 out of 10 FC samples (Supplementary Table S9).

### Exome sequencing data analysis and CNV calling

Raw data from exome sequencing was checked for per base quality score and reads having 80% bases with phred quality score 30 and greater were carried forward for downstream analysis and rest were discarded. Filtered reads were aligned to the reference genome (hg19) using BWA (version 0.6.1)^35^ allowing for 2 mismatches. More than 98% percent of the data was aligned to reference for each sample. Data was also checked for PCR duplicates and the same were removed. Aligned reads were used to call CNVs using *CoNIFER* (version 0.2.2)^36^ with default parameters.

### Genome-wide genotyping and CNV calling

Isolation of the genomic DNA was done using a standard salting-out procedure to perform genome-wide genotyping by using the Infinium Human660W-quad BeadChip method (Illumina, Inc., San Diego, CA, USA). We used 200 ng of genomic DNA for each sample, in accordance with the manufacturer’s guidelines. The raw data files were processed by the GenomeStudio software package. To call CNVs, we used the PennCNV algorithm and applied a stringent criterion of at least 10 consecutive probes to show altered intensity to qualify as a CNV call. We used a threshold of 0.35 for the standard deviation for logR ratio of normalized intensity (LRR) and a threshold of 0.05 for the standard deviation for B allele frequency as explained in our earlier study.

In order to confirm their genomic evidences for fusion events, we overlaid CNV coordinates obtained from above analysis with fusion breakpoints ( ± 500 Kb).

### Sequence enrichment around fusion transcripts

Nucleotide sequences extracted from 50 bases up-stream (from 5’ gene) and down-stream (from 3’ gene) around the fusion breakpoint were tested for any sequence preference. This part of analysis was performed using an in-house Perl script along with an online tool WebLogo to calculate the frequency of each base around the junction. (http://weblogo.berkeley.edu/logo.cgi).

### Chromosome proximity and fusion transcripts

To determine the chromosome proximity we used Hi-C data from ENCODE phase 3 experiments for ten different cell lines as explained in Supplementary Table S9. Paired-end Hi-C sequencing data were mapped and curated using a published pipeline HiCUP^37^ (v0.5.8) and Bowtie^38^ (v2.2.6). All sequencing data was mapped to hg19 reference genome at a resolution of 40 kb. A di-tag was considered only if it recurred in ≥ 2 cell lines with MAPQ ≥ 30 for both mates with Z score > 1.96. In order to check if there is any association of chromosomal proximity with fusion transcripts, we overlaid the identified chromatin map with these fusion breakpoints.

### In-silico translation and proteomics data analysis

To check the translational evidence for identified fusions, MS/MS Spectral datasets from human cortex were considered from PRIDE projects PXD000263, PXD004076, PXD004987, PXD005629 and PXD002775. These were downloaded from the PRIDE repository and were searched against the six frame translated database using OMSSA^39^ and X!Tandem^40,41^ in integrated transcriptomic-proteomic pipeline EuGenoSuite^42^.

We performed in-silico trypsin digestion for our probable peptides with one miss-matched site. Further parameters involve 20 ppm precursor ion tolerance; 0.5 Da product ion tolerance; carbamidomethylation of cysteine as fixed modification; and oxidation of methionine and peptide N-terminal acetylation were used as variable modifications. Stringent FDR threshold of ≤ 1% was applied to the resulting PSMs and junction specific peptides were identified using a customized Perl script. Reading frames for these filtered hits were further confirmed by again mapping them with human proteome (RefSeq GRCh37.p13) using stand-alone BLAST tool^43^.

### Gene and pathway enrichment analysis

To understand the functional significance of genes harboring fusions, pathway analysis was performed using Gene Set Enrichment Analysis (GSEA)^44,45^. We applied gene ontology enrichment analysis using KEGG pathways to generate gene clusters enriching similar biological processes. Gene cluster and their corresponding pathways with highest enrichment score and FDR q-value < 0.05 were only considered for literature mining.

### Statistical analysis

Differential numbers of fusion transcripts between groups of samples were screened out using Wilcoxon rank-sum test. P < 0.05 was considered as statistically significant. Inter-individual differences between cells and tissues were quantified by using Principal Component Analyss and were performed in R platform (http://www.rproject.org) using rlog-transformed read count from whole transcriptome.

### Validation and quantification

cDNA conversion of the extracted RNA (1 micro-gram) was done using High-Capacity cDNA Reverse transcription kit (Thermo Fischer Scientific) as per manufacturer’s protocol in a reaction volume of 20 µl. To target fusion junction we designed fusion specific primers by using Primer3 software (http://frodo.wi.mit.edu/primer3/) and their genomic locations were confirmed by UCSC’s In-silico PCR option. The list of primers used in this study is provided in Supplementary Table S10. These primers were subjected to PCR and to amplify the junction specific PCR product. Quantitative PCR (qPCR) was carried out using SYBR Green master-mix (KAPA) on Roche LC480 system using primers detailed in Supplementary Table S10. Fusion expression was determined using the delta-delta CT method^46^ and using B2M as the house-keeping gene.

## Supplementary information

Supplementary figures.

Supplementary dataset 1-10.
