# Supplementary material for: Fusion transcripts in normal human cortex increase with age and show distinct genomic features for single cells and tissues
Source: Sci Rep. 2020 Jan 28;10:1368. doi: 10.1038/s41598-020-58165-6 (PMC6987184; doi:10.1038/s41598-020-58165-6)
Supplement: Supplementary file 1 — Supplementary figures. [file 41598_2020_58165_MOESM1_ESM.pdf]

Fusion transcripts in normal human cortex increase with age and show distinct genomic features for single cells and tissues

Bharati Mehani[1,2], Kiran Narta[1,2], Deepanjan Paul[1,2], Anurag Raj[2,3], Deepak Kumar[1,4], Anchal Sharma[1,2], Lalit Kaurani[1], Subhashree Nayak[5], Debasis Dash[2,3], Ashish Suri[6], Chitra Sarkar[5], Arijit Mukhopadhyay*[2,7]

[1] Genomics and Molecular Medicine Unit, CSIR-Institute of Genomics and Integrative Biology, Mathura Road, New Delhi 110020, India

[2] Academy of Scientific and Innovative Research (AcSIR), Delhi, India.

[3] G.N. Ramachandran Knowledge Center for Genome Informatics, CSIR-Institute of Genomics and Integrative Biology, Mathura Road, New Delhi 110020, India,

[4] Department of Neurology, Neuroscience Centre, All India Institute of Medical Sciences, New Delhi, India 110029

[5] Department of Pathology, All India Institute of Medical Sciences, New Delhi 110029, India

[6] Department of Neurosurgery, All India Institute of Medical Sciences, New Delhi 110029, India

[7] Translational Medicine Laboratory, Biomedical Research Centre, University of Salford, United Kingdom, M5 4WT

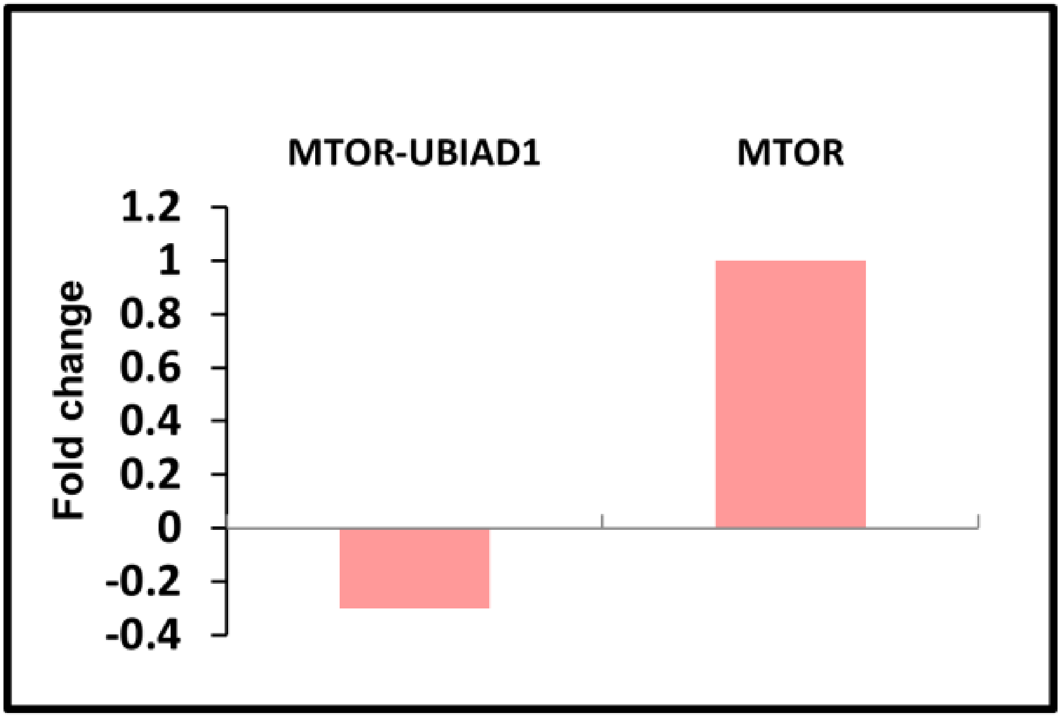

Supplementary Figure S1: Real time PCR for *MTOR-UBIAD1* fusion transcript showed down regulation with respect to its parent gene i.e. *MTOR* in FC171.

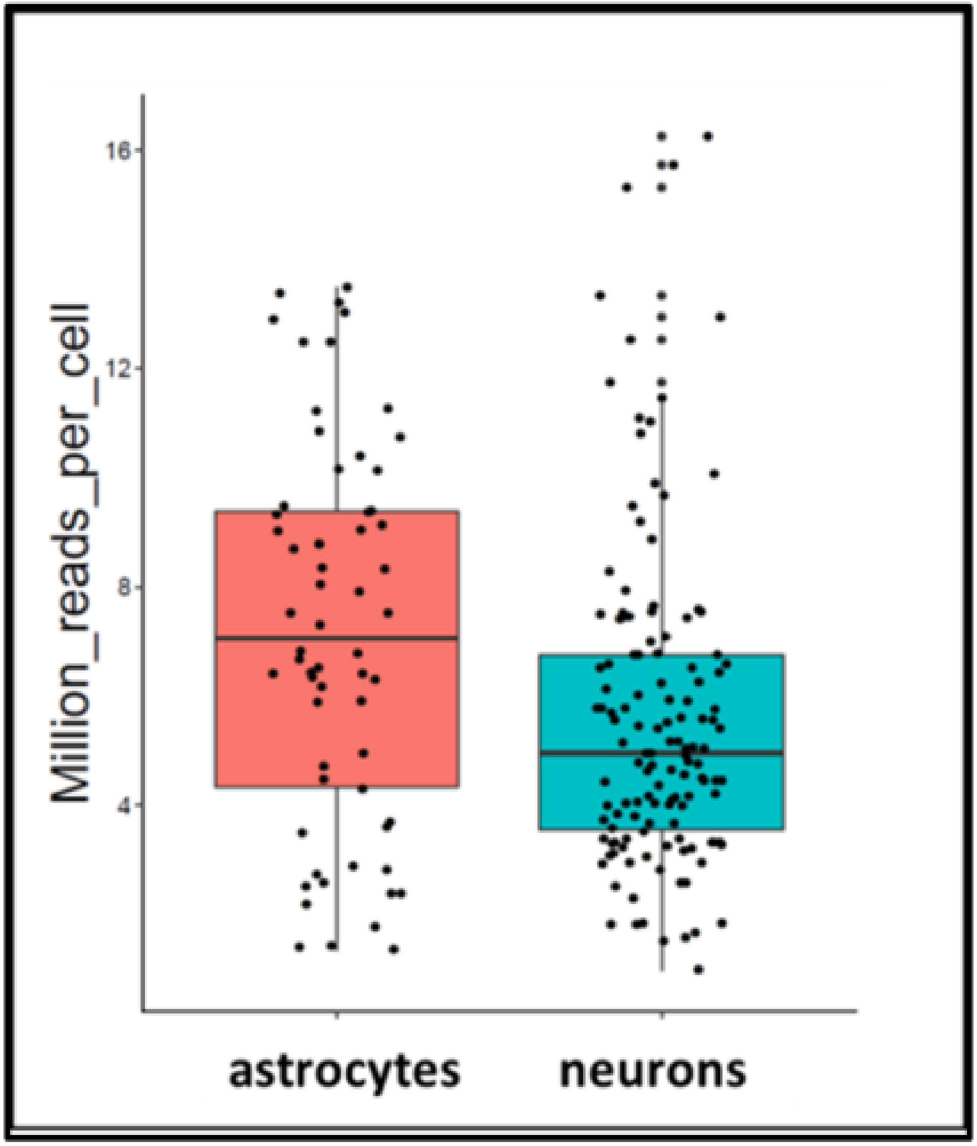

Supplementary Figure S2: Boxplot representing distribution of quality filtered read (million) per cell. Horizontal axis denotes brain cells while vertical axis represents million reads per cell. Cells with minimum 1 million reads were only considered and subjected to downstream analysis.

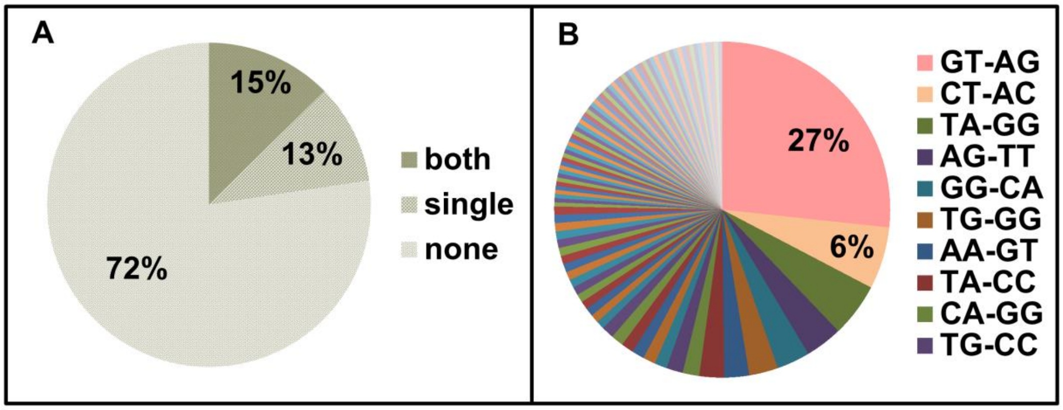

Supplementary Figure S3: Sequential context around fusion breakpoint identified in brain cells. (A) Percentage of fusion breakpoints that occur at the exon boundaries of one, both or neither of the genes that comprise fusion. (B) Percentages of fusion transcripts that use canonical splice site signature GT-AG, CT-AC, or another donor-acceptor sequential preference in brain cells.

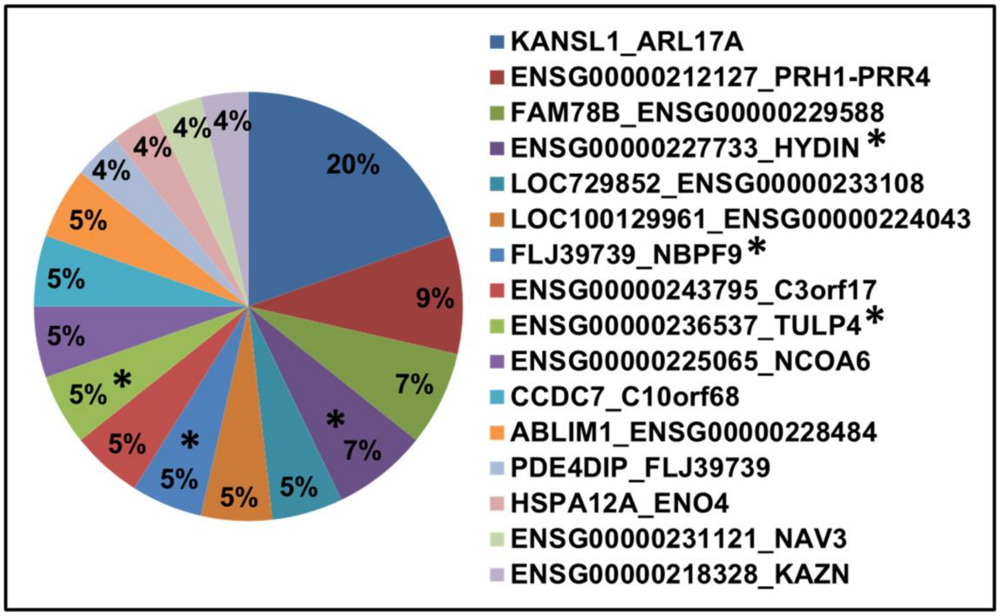

Supplementary Figure S4: Pie chart represents recurrent fusion transcripts identified in human brain. Each slice of the chart denotes each event and its recurring frequency.

Events marked with star are also identified in single neuron but no recurrent event was found in single astrocytes

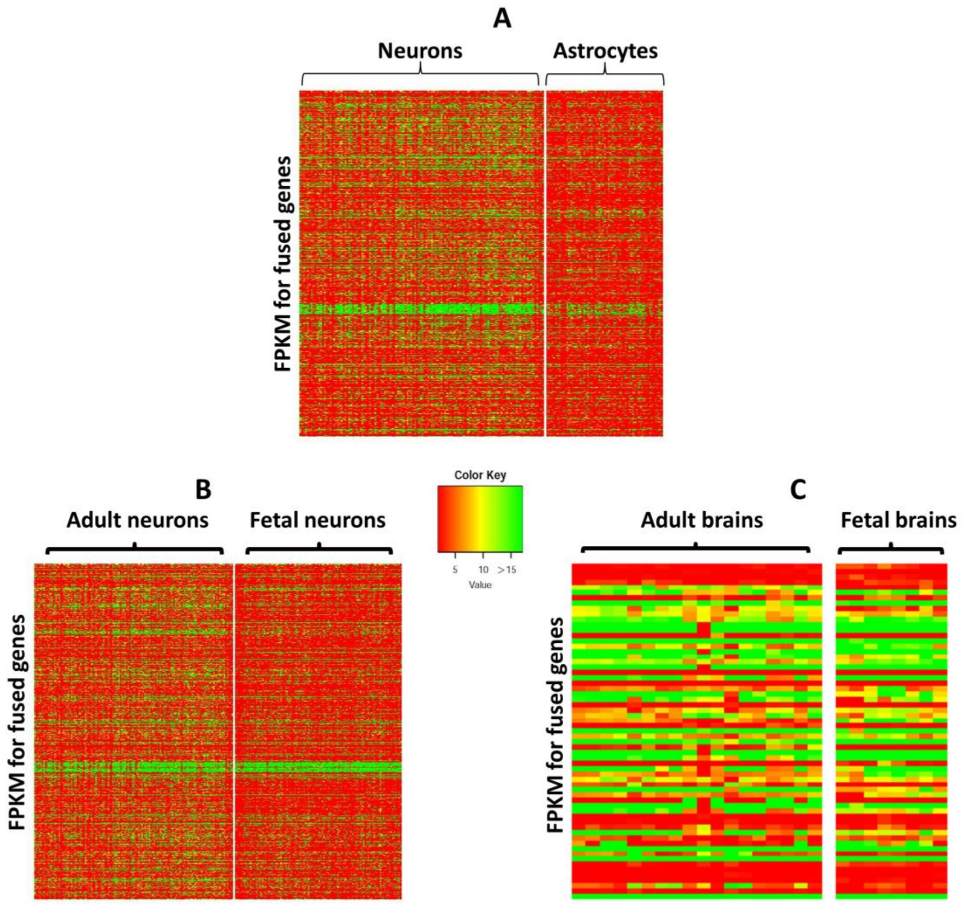

Supplementary Figure S5: Heatmap representing genes that are fused in (A) neurons (panel 1) have a considerable expression (FPKM >1) in astrocytes (panel 2). Similarly, the heatmap in its lower panel represents genes that are fused in (B) single cells and (C) tissues from adult brain (panel 1) have a considerable expression (FPKM >1) in fetal brain (panel 2). In all of these cases column represents each cell, and rows represent each gene. Color scale ranging from red indicating low expression values, yellow indicating intermediately expressed genes, to green representing highly expressed genes.

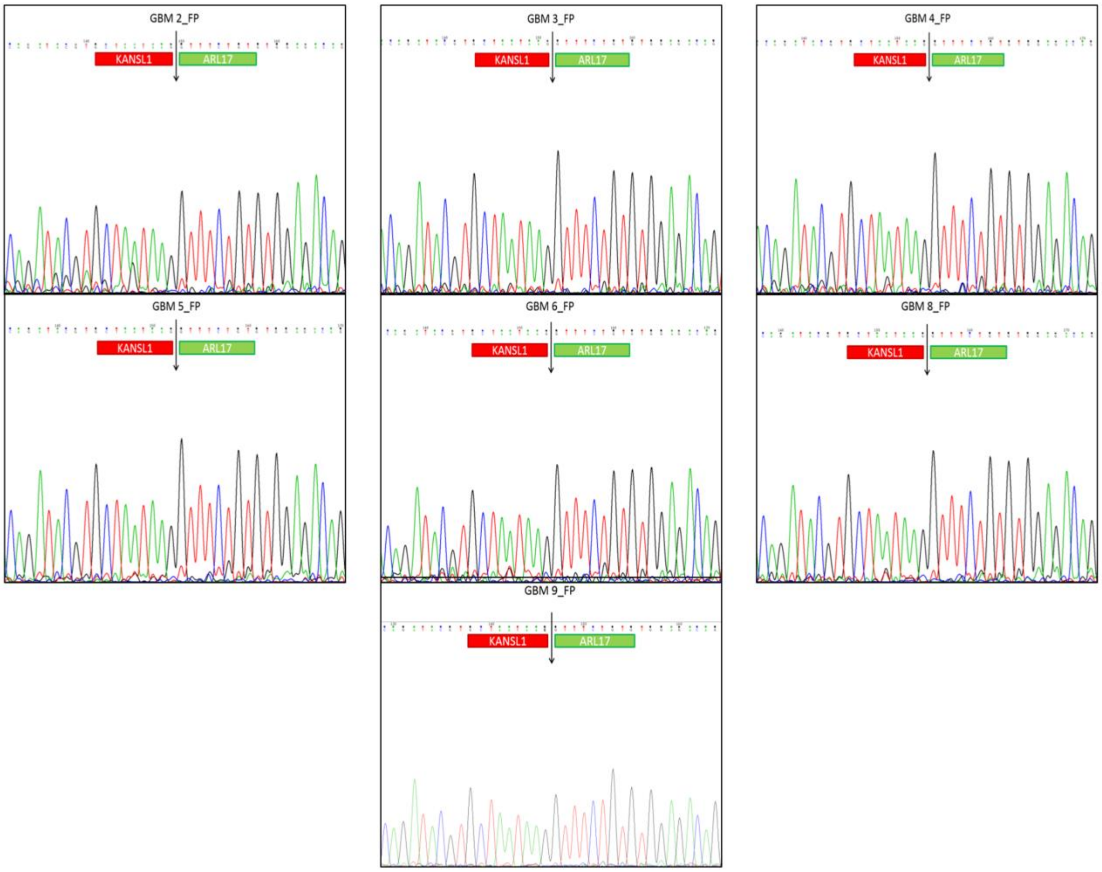

Supplementary Figure S6: Conformation of KANSL1-ARL17 fusion transcript in all 7 GBM samples by performing Sanger sequencing of the PCR product from fusion specific PCR

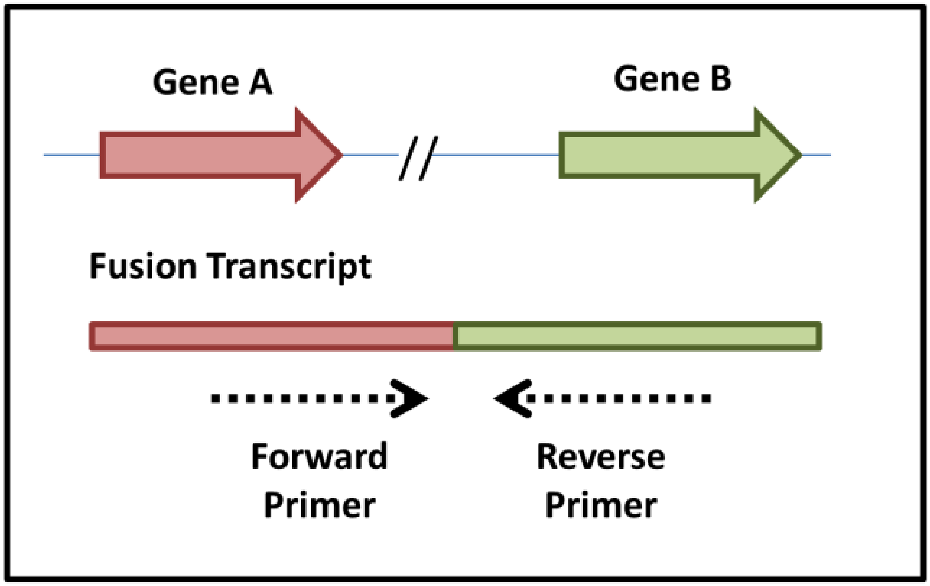

Supplementary Figure S7: Schematic representation for the strategy used for primer designing. Fusion specific primers targeting chimeric junction, were designed in such a way where forward primer would bind to gene A and reverse with gene B. When subjected to PCR will only amplify the junction specific PCR product.

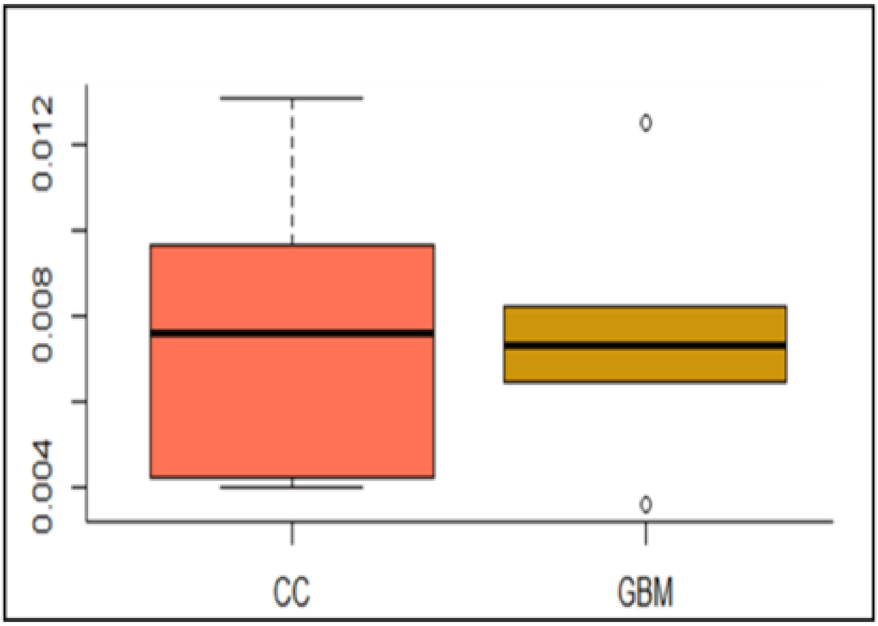

Supplementary Figure S8: Results from real time PCR using ARL17 primers with cDNAs from 6 in-house normal brains along with 6 GBM samples
